# Online Group–Based Dual-Task Training to Improve Cognitive Function of Community-Dwelling Older Adults: Randomized Controlled Feasibility Study

**DOI:** 10.2196/67267

**Published:** 2025-05-16

**Authors:** Pui Hing Chau, Denise Shuk Ting Cheung, Jojo Yan Yan Kwok, Wai Chi Chan, Doris Sau Fung Yu

**Affiliations:** 1 School of Nursing The University of Hong Kong Hong Kong China (Hong Kong); 2 Department of Psychiatry The University of Hong Kong Hong Kong China (Hong Kong)

**Keywords:** cognitive training, dual-task, co-design, online, older adults, Hong Kong

## Abstract

**Background:**

Cognitive training for older adults is crucial before cognitive impairment emerges. During periods of social distancing like the COVID-19 pandemic, cognitive stimuli are lacking. Online dual-task training is proposed as a solution to address these needs.

**Objective:**

We aimed to explore the feasibility, acceptance, and potential effects of online group-based dual-task training as an intervention for enhancing cognitive function among community-dwelling older adults.

**Methods:**

A randomized controlled feasibility study was conducted with 76 participants in Hong Kong, randomly assigned to the intervention and attention control groups in a ratio of 2:1 (n=50, 66% and n=26, 34%, respectively). The intervention group underwent 60-minute online dual-task training sessions twice a week for 12 weeks, incorporating cognitive components (upper limb and finger movement, arithmetic operation, and verbal fluency) and physical components (chair-based exercises) developed through a co-design approach. The attention control group received online health talks. Outcomes related to feasibility and acceptance included class attendance and self-reported satisfaction. Main outcomes related to potential effects included the Memory Inventory in Chinese and the Montreal Cognitive Assessment 5 Minutes (Hong Kong Version) at baseline, 6 weeks (midintervention), 12 weeks (postintervention) and 18 weeks (follow-up). Descriptive statistics and linear mixed effects models were used. Effect size was described with Cohen *d*. Qualitative feedback was collected from 12 informants and analyzed by thematic analysis.

**Results:**

About 72% (36/50) of the participants in the intervention group and 62% (16/26) in the control group attended over 75% of the classes. In total, 44 (88%) participants from the intervention group provided acceptance feedback; 82% (36/44) were satisfied and 84% (37/44) would recommend the training to others. Improvement in the Memory Inventory in Chinese score in the intervention group was observed at midintervention, postintervention, and follow-up, with a medium-to-large effect size (*d*=0.65, 0.43 and 0.85, respectively). Adjusting for baseline values, the between-group differences in the Montreal Cognitive Assessment 5 Minutes (Hong Kong Version) score attained a small-to-medium effect size at midintervention (*d*=0.34) and postintervention (*d*=0.23). Qualitative feedback highlighted the timesaving and convenient aspects of online dual-task training, with participants finding the sessions challenging and enjoyable, and reporting benefits across cognitive, physical, and psychosocial domains. However, a preference for traditional in-person training was noted among the older adults despite the advantages of online training.

**Conclusions:**

Online dual-task training is a feasible intervention accepted by the older adults, with potential benefits in cognitive abilities. Online training may complement in-person sessions. Further investigation in a full-scale randomized controlled trial is warranted to comprehensively explore its effects and address areas for improvement.

**Trial Registration:**

ClinicalTrials.gov NCT05573646; https://clinicaltrials.gov/study/NCT05573646

## Introduction

### Cognitive Decline

The cognitive health of older people is critical to aging in place. Even among healthy older adults, about one-third may experience cognitive decline within 18 months [1], impacting decision-making and daily activities like managing finances, medications, transportation, and meal preparation [2]. Cognitive decline is also linked to decreased physical performance and psychosocial issues, such as loneliness and depression [3]. The health care burden may thus increase in the long run [3].

### Existing Dual-Task Interventions

Interventions to enhance cognitive function or to prevent decline are essential for healthy aging. When older adults have limited capacity in performing both functional tasks and cognitive tasks, they tend to prioritize the former over the latter because of the lower chance of being injured [4]. Dual-task training, which involves combining cognitive and physical training components, has been shown to improve physical and cognitive performance, including, but not limited to, gait, balance, and memory and cognitive function [5]. Compared with training with single components, dual-task training tends to have potentially greater benefits [6,7]. Dual-task training has been widely applied among populations with clinical conditions, such as stroke, Parkinson disease, and cognitive impairment. A systematic review of 13 randomized controlled trials (RCTs) on stroke survivors reported that dual-task training could improve walking and balance function [8]. A recent meta-analysis of 14 RCTs estimated a small-to-moderate effect in improving cognitive function among stroke survivors [9]. Meanwhile, a meta-analysis of 17 RCTs on people with Parkinson disease revealed positive effects in physical performance, such as gait and balance, with effect size varying from small to large [10], and a narrative review of 3 studies reported potential improvements in cognitive function among this group, although statistical significance was not always achieved [11]. A 2022 network meta-analysis concluded that dual-task training was promising for addressing the cognitive and motor symptoms of patients with Parkinson disease [12]. The benefits of dual-task training for cognitive function were commonly investigated among cognitively impaired people. A review of 18 RCTs on those with dementia or mild cognitive impairment reported improvements in overall cognitive function, attention, and functional mobility [7]. Another meta-analysis of 21 RCTs reported small-to-moderate effects in global cognitive function, memory, executive function, and attention in cognitively impaired people [13]. A recent meta-analysis of 20 RCTs also reported benefits for global cognition, executive function, and working memory with effect sizes that were moderate, small to moderate, and moderate to large, respectively [14]. There is growing literature on studies targeting cognitively healthy older people. A meta-analysis of 8 controlled trials on cognitively healthy older adults reported improvement in cognitive functions, such as global cognition, working memory, and executive function [15]. The same review also reported no statistically significant difference in efficacy between cognitively healthy older adults and those with mild cognitive impairment [15].

### Service Gaps

In practice, motivating healthy older adults to engage in cognitive training can be challenging, especially when they perceive no immediate risk of decline. Presenting interventions as recreational and stimulating can enhance participation. Compared with solely cognitive training, dual-task training may address this need. Meanwhile, when there was social distancing, such as during the COVID-19 pandemic, older adults lost their opportunity to exercise and interact with others. Reductions in physical activity and social interaction not only challenged their physical and psychological health but also challenged their cognitive performance. Although group-based dual-task training could be beneficial, existing dual-task training programs have been conducted face-to-face, which limits their application during times of social distancing, prompting the exploration of online delivery methods. As the participants could join the training in their own homes, the training components had to be specially designed to simultaneously ensure safety, enjoyment, and efficacy. Hence, as an initial step, the feasibility, acceptability, and potential effects of the proposed intervention had to be investigated through a feasibility study.

### Aim and Objectives

This study aimed to explore the feasibility, acceptance, and potential effects of online group-based dual-task training as an intervention for enhancing cognitive function among community-dwelling older adults. To accomplish this aim, we had three objectives: (1) to develop the intervention through a co-design approach, (2) to explore its feasibility and acceptance among older adults, and (3) to examine its potential effects in terms of the cognitive, physical, and psychosocial status of the intervention group before and after the intervention and in comparison with the control group. We hypothesized that online based dual-task training would be feasible for healthy older adults.

## Methods

### Study Design

This was a randomized controlled feasibility study with 2 parallel arms. The trial was registered at ClinicalTrials.gov (NCT05573646).

### Ethical Considerations

Ethics approval was obtained from The University of Hong Kong/Hospital Authority Hong Kong West Cluster Institutional Review Board (UW22-038).

### Setting

This study was conducted in both online and community settings across Hong Kong. Data collection was conducted at older adults community centers, an older adult educational center, and a university campus, and the intervention was delivered online.

### Target Population and Sample

The target population comprised community-dwelling older adults. The inclusion criteria were (1) age ≥65 years, (2) no communication problems, and (3) ability to use an online meeting platform. The exclusion criteria were (1) contraindications to chair-based exercises and (2) engagement in any kind of cognitive training 3 months before the study or during the study period.

According to the literature, for a main trial designed with 80% power and 2-sided 5% significance to detect a small-to-medium effect (Cohen *d* between 0.1 and 0.3), a sample size of at least 20 per treatment arm is needed for a pilot study [16]. Meanwhile, to allow the sample to detect a medium within-group effect size in the intervention group (*d*=0.5) with 80% power and 2-sided 5% significance, a sample size of 34 was required for the intervention group. Taking both scenarios and 20% attrition into account, 50 participants in the intervention group and 25 participants in the control group were required.

### Intervention: Online Group-Based Dual-Task Training

Participants in the intervention group received a 1-hour online group-based dual-task training session twice a week for 12 weeks, resulting in a total intervention time of 24 hours. Such frequency and duration were within the range of 4 to 25 weeks and 30 to 240 minutes per week, as used in previous dual-task training programs for healthy older adults [6].

**Figure 1:**
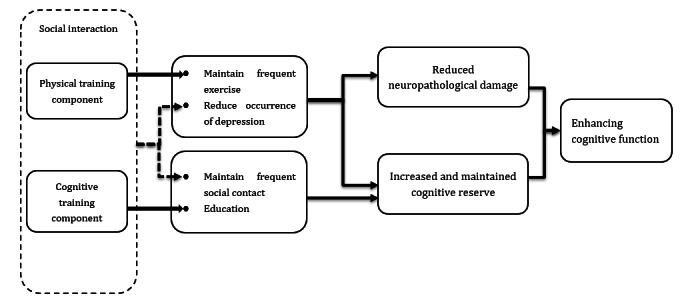
Conceptual framework.

Figure 1 shows the conceptual framework underpinning the development of the intervention based on the 2020 Report of the Lancet Commission on dementia prevention, intervention, and care [17]. The framework proposed that modifiable risk factors prevent dementia through reduction of neuropathological damage and increase and maintenance of a cognitive reserve. Among these factors, maintenance of frequent exercise and reduction of depression act on both paths, while maintenance of frequent social contact and education act on the cognitive reserve path. In our intervention, the physical training components helped to maintain frequent exercise (at least twice weekly) and the cognitive training component mimicked the education effect. The social interaction via group Zoom (Zoom Communications, Inc) classes maintained frequent social contact for at least twice a week and thus could help to relieve depression symptoms.

In the development of the cognitive training component, various factors were considered, including the lower education level of the local older adults, the online delivery format of the intervention, the cultural appeal to the local older population, and the effectiveness and safety of the training. A diverse set of training components were incorporated, such as upper limb and finger movements, arithmetic operations, and verbal fluency exercises, based on their proven efficacy in previous dual-task training studies [18,19]. A co-design approach was adopted to develop the dual-task training components. In total, 9 participants from the target population were invited to a trial run to provide feedback on their interests, challenges, and suggestions for tasks they preferred. The suggested tasks were then reviewed by the project team to assess their intensity and potential impact on training outcomes. Considering what would be acceptable and effective, 18 upper limb and finger movement games, 22 arithmetic operation games, and 6 verbal fluency training components were finalized (Textbox 1). During the 1-hour training session, participants engaged in different tasks according to a predefined schedule (Table 1). Specifically, participants spent 12 minutes on upper limb and finger movement exercises, 15 minutes on arithmetic operations, and 15 minutes on verbal fluency tasks. Each game started at the easiest level, with the facilitator adjusting the cognitive demand as participants progressed. For each game, the facilitator would start with the easiest level first. To sustain motivation and enjoyment, the difficulty level of each component increased when participants demonstrated mastery, typically indicated by 3 consecutive rounds without losing a game.

**Examples of cognitive training components.**

**Upper limb and finger movements**

- Goal-directed finger movements, such as counting and reverse counting with the fingers and making motions in different directions with different hands
- Forming patterns in the air

**Arithmetic operation**

- Counting
- Reverse counting
- Counting or reverse counting with subtraction or addition
- Clapping at specific numbers or multiples

**Verbal fluency**

- “Jie Long” (a well-known Chinese word chain game)
- Naming games (particularly on Chinese cultural concepts, such as Chinese dim sum and greetings in the Chinese new year)
- Memory games (participants needed to recap the previous terms presented by others and then add their own)

**Table 1:** Dual-task training schedule for each 60-minute session.

Time (minutes)	Dual-task training	
	Physical components (chair-based)	Cognitive components
5	Warm-up exercise	Safety reminder or casual chit-chat
3	Toes raises	Upper limb and finger movement 1
3	Heel raises	Upper limb and finger movement 2
3	Toes raises	Upper limb and finger movement 3
3	Heel raises	Upper limb and finger movement 4
3	Break	Break
3	Stepping	Arithmetic operation 1
3	Rest	Arithmetic operation 2
3	Stepping	Arithmetic operation 3
3	Rest	Arithmetic operation 4
3	Stepping	Arithmetic operation 5
3	Break	Break
3	Stepping	Verbal fluency 1
3	Clam exercise	Verbal fluency 2
3	Stepping	Verbal fluency 3
3	Clam exercise	Verbal fluency 4
3	Stepping	Verbal fluency 5
3	Clam exercise	Verbal fluency 6
4	Cool-down exercise	Safety reminder

For the physical training component, we selected chair-based exercises, which were chosen due to their suitability for the limited space available in most local households. Chair-based exercises offer various benefits, such as improving mood and well-being, enhancing certain activities of daily living, promoting social interaction, and increasing muscle strength [20]. Alongside the cognitive training games, participants engaged in exercises like toe or heel raises on alternate sides, stepping, and clam exercises (Table 1). The exercises were performed at a low frequency, such as 60 steps per minute. To ensure safety, participants were permitted to use their hands for balance support during stepping or clam exercises. Therefore, when upper limb and finger movement tasks were involved, only toe or heels raise were performed.

A group-based training approach was adopted as group-based cognitive training might yield greater interaction and cognitive benefits than individual-based training [21]. Group-based exercise was also reported as having the additional advantage of creating a social support environment that could enhance psychosocial well-being [22]. Moreover, group-based interventions tend to have higher adherence rates than general exercise programs due to the motivational support provided by group dynamics [23]. To encourage interaction among participants and enable close monitoring by the facilitator, a group size of less than 10 people was used.

Zoom was selected as the training platform due to its widespread use and familiarity among local older adults. Throughout the session, participants were instructed to sit on a sturdy chair with a back rest in front of a desktop computer, laptop, tablet, or mobile phone securely placed on a table. Mobile device users were provided with stands for better posture (all participants received the stand as a souvenir). To allow enough space for movement, the chair and table needed to be positioned 60 cm apart. Participants were encouraged to turn on their cameras during the session to facilitate interaction with one another. They were also asked to keep their audio on to actively participate in the cognitive tasks by verbalizing their answers.

The sessions were led by a trained facilitator. The facilitator did not need to be health professional, but they were required to undergo necessary training to ensure they had the knowledge and skills to effectively work with older adults [20]. A written manual with all the rules and regulations of the training was provided. During the initial sessions of the intervention, the project lead provided on-site supervision of the facilitator to ensure that the intervention was delivered according to the protocol. In addition, random spot checks were conducted by the project lead to ensure strict adherence to the protocol.

### Attention Control: Interactive Health Talks

In line with recommendations for attention control groups [24], participants in the control group underwent 8 one-hour online group-based interactive health talks on food label knowledge and its practical applications. These sessions were led by a facilitator with nutrition training.

### Outcome Measures

The primary outcomes were related to feasibility and acceptance. Feasibility was reflected by class attendance. Reasons for absence were documented when available. There was no consensus on the definition of high attendance [25]. We took 75% attendance as high attendance, as a review reported 3 of 11 studies on exercise interventions adopted this definition [25]. The proportion of participants with high attendance was calculated using the number of participants randomized to the group as denominator. A review of group exercise interventions for older adults reported that in 4 of 6 studies, 65% to 67% of participants adhered to the program [22]. Hence, our study considered over 65% of participants completing at least 75% of the classes as feasible.

Acceptance was reflected by 5-point Likert scale questions on the level of satisfaction with the intervention (“Are you satisfied with this training?”) and the level of likeliness of recommending the intervention to other people (“Would you recommend this training to friends and family”), with answers collected at the end of the intervention via instant text messaging. The proportion of respondents providing a positive response was calculated. As a conservative estimate, another proportion was calculated by assuming those who did not provide feedback as not being satisfied and not likely to recommend the intervention, with the number of participants randomized to the group as the denominator.

The secondary outcomes were related to potential effects, which included subjective memory complaints, cognitive status, working memory, executive function, physical function, instrumental activities of daily living, happiness, and social networks.

Subjective memory complaints were assessed using the Memory Inventory in Chinese (MIC) [26]. This scale consists of 27 items on memory concerns related to daily activities in the past month. Responses are rated on a scale from 0 (none) to 4 (once or more per day or continuously), with scores totaling between 0 and 108. A higher score indicates a greater frequency of memory concerns.

Cognitive status was assessed using the validated Montreal Cognitive Assessment 5 Minutes (Hong Kong Version) (HK-MoCA 5-Min), which covers 4 domains, namely, attention, executive functions or language, orientation, and memory [27]. The total score ranges from 0 to 30, with a higher score indicating better cognitive status.

Working memory was assessed by Digit Span Test [28]. Participants were required to repeat progressively longer series of digits. The test began with 2 digits and increased in length, with a maximum of 8 digits for the forward test and 7 digits for the backward test. The participant’s ability to recall the longest digit series in forward and backward order was recorded as the forward and backward scores, respectively. A higher score indicated a better working memory capacity.

Executive function was assessed by the Chinese version of the Victoria Stroop Test [29]. The test consisted of 3 parts, each presenting different stimuli: colored dots, common words unrelated to color, and color words. Participants were required to name the color in which the stimuli were printed. Inference scores were calculated based on the difference in completion time between the word or color test and the dot test, with a longer completion time indicating a poorer condition.

Lower-limb muscle strength was assessed by the 5-time chair stand task [30]. Participants were timed on how long it took them to complete the task, with a longer time indicating poorer performance. If participants were unable to finish the task within 1 minute, the test was stopped, and a time of 60 seconds was imputed for analysis purposes.

Instrumental activities of daily living were assessed by Lawton’s 8 selected tasks, which include telephone use, shopping, food preparation, housekeeping, laundry, transportation, medicine use, and handling finances [31]. Participants rated their level of dependence in performing each task on a 5-point Likert scale. The score was the sum of all items, ranging from 0 to 8, with a higher score indicating greater independence.

Happiness was assessed by the Chinese version of the validated 4-item Subjective Happiness Scale [32]. Participants rated their responses on a 7-point Likert scale. The score was the average of the 4 items, ranging from 1 to 7, with a higher score indicating a higher level of happiness.

Depressive symptoms were assessed by the Patient Health Questionnaire-9 (PHQ-9) [33]. PHQ-9 was validated as a reliable tool for evaluating depression among older adults. The scale ranged from 0 to 27, with a higher score indicating a higher level of depressive symptoms.

Social networks were assessed by the Chinese version of the 6-item Lubben Social Network Scale [34]. Participants rated their responses on a 5-point Likert scale. The total score ranged from 0 to 30, with a higher score indicating a stronger social network.

All effect-related outcomes were assessed at recruitment (T0, baseline), 6 weeks after baseline (T1, midintervention assessment), 12 weeks after baseline (T2, postintervention assessment), and 18 weeks after baseline (T3, follow-up assessment). Demographic, medical and lifestyle information, such as age, gender, education level, exercise habits (measured by the International Physical Activity Questionnaire-Short Form), frailty status (measured by the FRAIL scale, which consists of 5 items, namely, fatigue, resistance, ambulation, illnesses, and loss of weight) and comorbidity (coexistence of 2 or more chronic illnesses) were collected at baseline. For participants who could not undergo face-to-face assessment for reasons such as quarantine or sickness, the 5-time chair stand task was not administered and was regarded as missing data.

Qualitative feedback was collected from a purposive sample at the end of the follow-up period through phone interviews. A semistructured interview guide was used to explore reasons for participating in the intervention, perceived difficulties encountered, logistical aspects of the intervention, and strengths and limitations perceived by participants. Prompts were provided as appropriate. Participants who exhibited varying degrees of change in the MIC score were invited to ensure diverse perspectives. Data saturation was achieved with 12 informants.

### Procedure

Participants were recruited through social media and nongovernmental organizations in Hong Kong. They were randomly assigned to the intervention or control groups at a ratio of 2:1. As a strategy to enhance recruitment, a ratio of 2:1 was proposed for the number of people in the intervention and control groups, such that less participants would be disappointed with the allocation to the control group. A face-to-face orientation session was conducted to guide participants on using the Zoom platform and provide them with mobile device stands. Furthermore, for intervention group participants, demonstrations of the chair-based exercises were provided as they were unable to observe lower-limb movements on Zoom.

### Randomization and Concealment

Block randomization with varying block sizes was used to generate the allocation sequence. An independent research assistant generated the sequence using an online platform and prepared sequentially numbered opaque sealed envelopes. These envelopes were opened in front of the participants immediately after obtaining informed consent.

### Blinding

This was a single-blinded study as participants and interventionists could not be blinded. Trained research assistants involved in assessing outcomes related to potential effects were blinded to the allocation.

### Data Analysis

The participants’ characteristics were summarized using descriptive statistics. Attendance statistics, regarding the percentage of scheduled classes attended, were calculated. The percentage of participants satisfied with the intervention and those who would recommend it to others were also calculated. Adopting intent-to-treat, linear mixed effects models were used to analyze effect-related outcomes, adjusting for sociodemographic characteristics, medical history, and lifestyle patterns. A significance level of 5% was used. However, given the small sample size, focus was placed on effect size with the corresponding 95% CI. A Cohen *d* of 0.2 was considered clinically meaningful [35]. Missing outcome measures were not imputed as the linear mixed effects models could handle missing data. Statistical analyses were performed using SPSS version 28.

As for the qualitative interview, the audio recordings were transcribed verbatim for thematic analysis [36]. First, 2 researchers read the transcripts several times and conducted the systematic coding independently. They proceeded to generate the initial codes and the recurrent pattern within the data and identify and name the themes and subthemes according to the underlying meaning. Then, a third researcher checked for the consensus on the themes and subthemes and discussed with the team accordingly. They also worked together to identify the common threads that extend across the interviews of the participants. Analyses were performed manually without the aid of software.

## Results

### Overview

**Figure 2:**
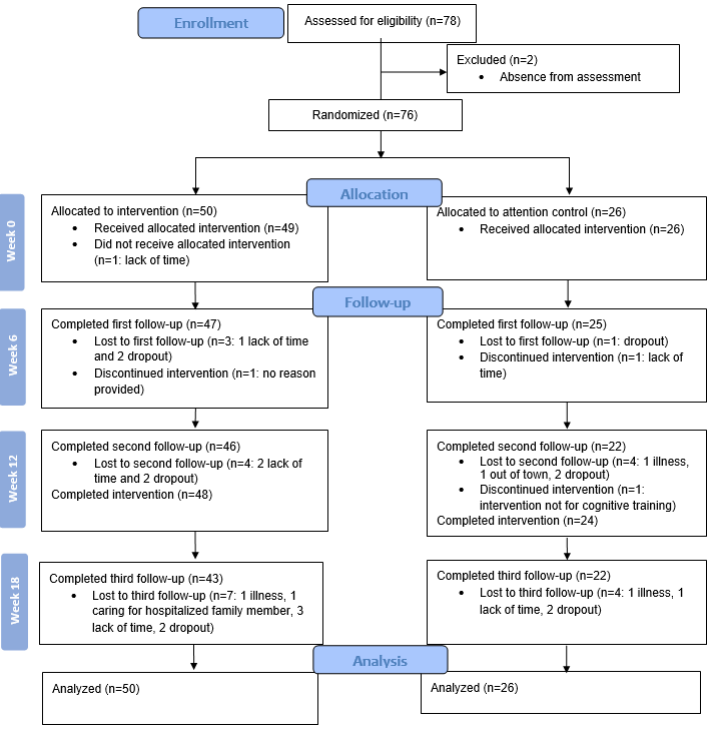
CONSORT (Consolidated Standards of Reporting Trials) diagram.

In total, 76 participants were recruited from October 2022 to May 2023 (Figure 2). To manage class sizes, the 50 participants in the intervention group were divided into 8 subgroups, each with 24 classes, and the 26 participants in the attention control group were divided into 2 subgroups, each with 8 classes. Overall, 48 (96%) participants from the intervention group and 24 (92%) from the control group completed the assigned intervention.

The mean ages of the participants in the intervention and the control groups were 71.5 (SD 5.2) and 71.1 (SD 6.0) years, respectively. There were 64% (32/50) female participants in the intervention group and 88% (23/26) in the control group. One-fifth (15/76, 20%) of the participants had a primary level of education or below. About 44% (22/50) of the participants in the intervention group and 54% (14/26) in the control group had multimorbidity. Most participants in both groups (30/50, 60% and 16/26, 61%) had low levels of physical activity. Prefrailty was observed in about 38% (19/50) of intervention-group participants and 50% (13/26) of control-group participants. Table 2 shows the characteristics of the participants.

**Table 2:** Baseline characteristics of participants (N=76).

Characteristics		Intervention (n=50)	Control (n=26)
Age (y), mean (SD)		71.5 (5.2)	71.1 (6.0)
**Sex, n (%)**			
	Male	18 (36)	3 (11)
	Female	32 (64)	23 (89)
**Education level, n (%)**			
	Primary or below	10 (20)	5 (19)
	Secondary	27 (54)	14 (54)
	Tertiary or above	13 (26)	7 (27)
**Multimorbidity^a^, n (%)**			
	No	28 (56)	12 (46)
	Yes	22 (44)	14 (54)
**Physical activity level (measured by IPAQ^b^), n (%)**			
	Low	30 (60)	16 (61)
	Moderate	15 (30)	9 (35)
	High	5 (10)	1 (4)
**Frailty status (measured by FRAIL^c^ scale), n (%)**			
	Robust	31 (62)	13 (50)
	Prefrail	19 (38)	13 (50)

**^a^**Multimorbidity is defined as the co-existence of at least 2 of the following long-term health conditions: hypertension, heart disease, high cholesterol, cancer, osteoporosis, and other chronic illnesses, as reported by the participants.

**^b^**IPAQ: International Physical Activity Questionnaire.

**^c^**FRAIL: Fatigue, Resistance, Ambulation, Illnesses, and Loss of weight.

### Feasibility

In the intervention group, 36 (72%) participants attended over 75% of the 24 classes. For the attention control group, 16 participants (61%) attended over 75% of the 8 classes. Common reasons for absence in both groups included time clashes with medical appointments, illness, or other prior commitments.

### Acceptability

Among 44 participants who provided acceptance feedback on the intervention, 82% (36/44) expressed being very satisfied or satisfied with the intervention, and 84% (37/44) indicated they would be very likely or likely to recommend the intervention to others. Assuming that those who did not provide feedback were not satisfied and were not likely to recommend the intervention, the satisfaction rate was 72% (36/50) and the recommendation rate was 74% (37/50).

### Potential Effects

Regarding the effect-related outcomes, owing to the pilot nature of the trial, statistical significance was not the primary focus, instead, the effect size was noted. Within-group changes were explored and are presented in Multimedia Appendix 1. For the intervention group, the effect size of the MIC improvements at midintervention, postintervention, and follow-up compared to baseline were 0.65, 0.43 and 0.85, whereas the estimates for the control group were 0.51, 0.37 and 0.78, respectively. Similarly, both groups experienced an increase in HK-MoCA 5-Min score with moderate to large effect sizes. However, changes in working memory, as assessed by the Digit Span Test, did not consistently show the same direction in both groups. The intervention group showed a moderate improvement in executive function, as shown by the inference score for color from the Victoria Stroop Test, while the control group showed a small-to-large improvement in the inference score for words. Improvement was not observed for other outcomes in both groups.

Between-group differences, adjusting for the baseline differences, were explored and are presented in Multimedia Appendix 1. The intervention group did not outperform the control group in the MIC, but it showed better performance in the HK-MoCA 5-Min at the midintervention and the postintervention assessments (effect size 0.34 and 0.23, respectively). Meanwhile, a medium effect size was observed for between-group difference in the Digit Span Test at the follow-up. However, the findings for the Victoria Stroop Test were inconsistent. Details on other outcomes are presented in Multimedia Appendix 1.

Comparing the effect-related outcomes between participants with high attendance (≥75% of class) and low attendance in the intervention group (Multimedia Appendix 1), those with high attendance had fewer subjective memory complaints than those with low attendance at midintervention and postintervention, with effect sizes of 1.18 and 1.53, respectively. At the follow-up assessment, the between-group difference was observed to be in favor of the high-attendance group in the Digit Span Test, inference score for color, and PHQ-9 score.

### Qualitative Feedback

Table 3 shows the demographic characteristics of the 12 informants. Table 4 shows the 5 identified themes and the corresponding subthemes derived from the qualitative feedback, along with the significant quotes. The themes are summarized as presented in Textbox 2.

**Table 3:** Characteristics of the 12 informants providing qualitative feedback.

Informant	Sex	Age group (y)	Education level
A	Female	65-74	Primary
B	Female	65-74	Secondary
C	Male	≥75	Primary
D	Female	65-74	Secondary
E	Female	65-74	Secondary
F	Female	65-74	Tertiary
G	Female	≥75	Secondary
H	Female	65-74	Secondary
I	Female	65-74	Secondary
J	Male	65-74	Tertiary
K	Male	65-74	Primary
L	Male	65-74	Tertiary

**Table 4:** Five identified themes and the corresponding subthemes from the qualitative feedback and the significant quotes.

Themes and subthemes			Significant quotes by informants
**Perceived strength of the training (before participation)**			
	Arousing curiosity and motivation to join	“I heard an introduction at the elderly community center, and I want to learn more.” [Informant A] “Curious about online dual task training.” [Informant B]	
	Giving them hope to slow down cognitive decline	“I am taking precautions because I don’t know if I will have cognitive impairment in the future. I just want to prevent it. Early prevention is better.” [Informant D] “I want to maintain, not improve, cognitive ability.” [Informant L]	
	Offering opportunity for social interaction	“I want to make new friends.” [Informant C]	
	Save travel time	“No need to spend time going back and forth; you can stay at home. Nowadays, we mostly go to centers to learn things. In-person is much better, but it takes more time to get there and back.” [Informant E] “But overall, I think online is good for participants, as it saves travel time.” [Informant J]	
**Positive experience from the training**			
	Comprehensive training	“I think the course is quite good and comprehensive; it does not just focus on those with cognitive impairment.” [Informant I]	
	Challenging training	“There is some difficulty; sometimes I forget, like moving a hand or getting distracted and forgetting to do the stepping. You need to stay focused to complete it.” [Informant B] “At the beginning, I feel a bit frustrated when playing. However, it has its benefits. If everything were easy, there would be no need for training.” [Informant G] “If you have to move both your foot and hand, you often end up neglecting one. Sometimes, you also have to keep track of numbers, so you need to pay attention to what the other participants are saying.” [Informant K]	
	Engaging facilitator	“The host guided us with patience.” [Informant A] “Overall, I think the team is very dedicated. Sometimes when we can’t hear, she [the facilitator] reminds us. Or when we can’t think of an answer, she gives us hints at the right time. I think the whole process is very smooth.” [Informant I]	
	Peer support and encouragement during the training	“We encourage each other during the training.” [Informant L]	
	Enjoy the convenience at home environment	“I can start the training right after breakfast. When we have breaks, we can go to the washroom and drink water at home. After the session ends, we can prepare lunch ourselves.” [Informant A]	
**Challenges encountered with online training**			
	Constrained supervision and intensity of training	“At least with Zoom, the camera cannot capture my feet, so you cannot tell if participants are doing stepping. I think in-person is ultimately more useful because you can see everyone’s movements.” [Informant B] “Because in-person, you can immediately see everyone’s performance.” [Informant D]	
	Harder to follow the sequence if the training involved taking turns	“Because it is online and most participants are women, it is hard for me to know that it is my turn unless there is a man preceding my turn. But after a few sessions, everyone starts to get familiar with each other, and we build up rapport about the sequence.” [Informant J]	
	Less concentrated and less cordial	“During training, some might not be able to concentrate, for example, if there are things happening at home.” [Informant A] “It’s not as easy to get distracted if it is in-person.” [Informant D]	
	Class atmosphere not comparable to in-person class	“But in-person is more enjoyable, with more group interactions, which makes it more fun.” [Informant G]	
	Technical issues encountered	“The display is limited in size, and there might be some lag time.” [Informant J] “The problem is that some resource-limited families don’t have Wi-Fi at home, so they have to go to the center to access it, which is less convenient.” [Informant K]	
**Perceived benefits of the training**			
	Improved cognitive ability (including attention and concentration)	“Initially, I felt very unfamiliar with it, but after a few more sessions, I absorbed new knowledge and it made my mind more agile.” [Informant G] “I think I can use my brain more, as I usually don’t use it much.” [Informant H] “My memory has been maintained without much decline.” [Informant L]	
	Improved coordination and physical flexibility	“After participating, my knowledge has broadened, and both my hands and mind have become more agile.” [Informant C] “I find that after training, the coordination of my hands and feet has become a bit more agile compared to before.” [Informant L]	
	Improved communication skills	“It can train an older person to handle different communication demands.” [Informant J]	
	Promotion of positive mood and social interactions	“Someone accompanies you. I also find it fun and enjoyable.” [Informant F]	
	Learn new skills for self-practice in future	“I have not experienced this kind of hand-eye coordination, but now I do. Sometimes, when I’m alone, I practise the movements.” [Informant E] “I’ll go back and practise it myself.” [Informant K]	
	Increased self-efficacy and self-confidence	“My brain is now willing to exercise and let me do calculations.” [Informant F] “I actually feel quite happy and it gives a sense of accomplishment because you can achieve your goals and meet the training requirements, so there is a sense of success.” [Informant J]	
	Self-explanation of less benefit from “cheating”	“I do not feel like I have gained much. Maybe I prepare the answers in advance. For example, knowing that the next lesson will involve ‘clapping on 7,’ I will prepare the answers beforehand. But if it is in-person, it is harder to cheat.” [Informant I]	
**Suggestions for improvements**			
	Continue the training (beyond 12 weeks)	“I think I need to keep doing it consistently to see improvement. But I understand it is difficult if resources are lacking. Once the training stops, like now, I have forgotten a lot.” [Informant K]	
	Booster training	“Suggest to add online virtual courses. Each session would be half an hour, allowing students to choose their own study times.” [Informant K] “If possible, it would be best to have a booster session once a month to refresh what we have learned.” [Informant L]	
	Consider in-person mode (but reduced frequency to accommodate time and venue constraints)	“I think in-person sessions are ultimately more useful because you can see everyone’s movements. In-person sessions could be held once a week, not too frequently, since not everyone can attend twice a week, and the center might not be able to provide the space for two days of training.” [Informant B]

**Summary of the themes and subthemes derived from qualitative feedback.**

- Perceived strength of the training (before participation): informants found dual-task training to be a novel approach, which interested them and motivated them to participate. They expected the training would help to slow down cognitive decline. The online format of the training saved them travel time but could still offer social interaction.
- Positive experience from the training: informants described the training comprehensive and challenging. The engaging facilitator increased their adherence. The group training enabled peer support, enhancing their training experience. Informants also found having the training at home very convenient.
- Challenges encountered with online training: supervision and the intensity of training was limited by the online format. Some informants found it harder to follow the sequence if the training involved taking turns. Some found the home environment made them less concentrated and the class atmosphere was not comparable to in-person class. The quality of the experience was also influenced by the display size of their device, Wi-Fi speed, and more importantly accessibility to a stable internet connection.
- Perceived benefits after the training: informants perceived benefits in cognitive ability, coordination, physical flexibility, communication skills, positive moods, and social interactions. Some valued acquiring new skills that could enable future self-practice. Some mentioned increased self-efficacy and self-confidence. Meanwhile, one mentioned the use of a cheat sheet as related to the negligible improvement.
- Suggestions for improvements: informants expressed a desire to continue the training by either extending the training period or offering regular booster sessions. However, informants generally preferred in-person training, although they acknowledged limitations regarding time and venue.

## Discussion

### Principal Findings

This study demonstrated feasibility and explored potential effects of an online dual-task training for community-dwelling older adults. The innovative aspects of the online dual-task training included (1) the delivery mode, allowing for uninterrupted training even during a pandemic, (2) chair-based exercises suitable for home-based practice, (3) cognitive training tailored to the needs of Chinese older adults with lower education levels, and (4) a co-design approach that considered the preferences and interests of the participants when designing the training components.

This study was launched at the end of 2022 when the COVID-19 pandemic was still ongoing. While there was no citywide quarantine policy in place, there was still a home quarantine policy for those who were infected and their close contacts. Older adults tended to limit their outings to reduce the risk of infection, as well as chance of becoming close contacts. The older population had experienced a lack of activities for a prolonged period, potentially leading to a faster decline in cognitive performance. The group-based online dual-task training addressed this gap by offering training that the participants could participate in at home.

The findings of our pilot study showed the intervention was highly feasible, as 72% (36/50) of the participants in the intervention group attended over three-quarters of the classes, which was above the target of 65%. The intervention was also well received by the participants; 82% (36/44) of the participants were satisfied with the intervention and 84% (37/44) would recommend the intervention to others. Even adopting the most conservative approach, such figures remained high at 72% (36/50) and 74 (37/50), respectively. The dual-task training successfully attracted some relatively cognitively healthy participants to join and could be continued irrespective of the pandemic situation. This initiative addressed the existing gap in services by providing online cognitive training for community-dwelling older adults.

Within-group improvement was observed in subjective memory complaints and cognitive function in the intervention group. Such improvements became evident at midintervention and persisted until the final follow-up, showing a medium-to-large effect size. Despite the qualitative feedback indicating self-perceived benefits, other outcomes did not consistently favor the intervention. Controlled for the baseline outcomes, small-to-medium effects ranging from 0.23 to 0.34 were observed in cognitive function for the between-group difference in midintervention and postintervention assessments. These findings are consistent with previous literature reporting small effects of 0.22 to 0.29 for dual-task training over control groups [37,38]. Nevertheless, inconsistent results were reported for other outcomes. Moreover, the fluctuation in the magnitude of the improvement was unexpected. Although possible self-practicing of the dual-task training after the intervention period might explain the increase in within-group improvement at the follow-up, the drop at the postintervention assessment was unexpected. There were factors that might have affected the evaluation. For example, engagement in the environment might influence achievement in relation to the effects of cognitive training [39], but the social context of the participants was not assessed. Another possibility might be COVID-19 infection, which might influence the performance of the participants not only during the infection period but afterward [40]. Moreover, some assessments could not be performed face-to-face but were conducted via Zoom; the different modes of data collection might have also influenced their performance. After the pandemic, a proper RCT should be conducted.

Meanwhile, within-group improvement was observed for the attention control group. The attention control condition consisted of a series of interactive health talks focusing on nutrition and food labeling. Participants memorized the contents delivered in each talk so they could answer the instructors’ questions. Moreover, some participants had to learn to operate the Zoom platform as it was new to them. The learning process might also be beneficial to the participants, as some calculation was involved in comparing food labels using different units. In future trials, usual care control may be adopted.

Qualitative feedback highlighted the strength of the training regarding convenience, innovation, and comprehensiveness. In contrast, participants expressed a preference for in-person interactions. Hence, online training could be considered as a potential alternative or complement to traditional in-person training. Participants engaged in the training before receiving a cognitive impairment diagnosis, indicating that dual-task training could motivate early engagement in cognitive training. The facilitator leading the training does not necessarily be a health professional [20], and it is the engaging nature of the training, as revealed from the feedback, that the participants highly valued. This makes implementation of the intervention more feasible. Apart from the cognitive training, the participants also treasured social interactions and peer support during the class, and these further support the need to organize group-based training.

### Implications

Dual-task training offers sufficient challenges to community-dwelling older adults; they found it interesting and reported that it boosted their motivation to participate in cognitive training. The contents of the training were comprehensive, covering motion, arithmetic operations, and verbal fluency. The training was conducted in a stimulating and recreational atmosphere, which was well received by the participants.

Online training offers the advantage of saving time and being convenient. However, it is not intended to replace traditional in-person training, as older adults generally prefer face-to-face interactions. Nonetheless, online training can be viewed as an alternative or complement to in-person sessions. Participants highlighted that organizing training twice a week in-person would be challenging, indicating a potential solution of combining one day of in-person training with one day of online training to maintain a twice-weekly training frequency. As older adults become more accustomed to online training, it can serve as a viable option during situations like a pandemic, enabling training to continue even when older adult community centers are closed or during citywide quarantine measures.

When considering the implementation of in-person or hybrid mode training, it is essential to conduct research studies to assess their effectiveness. If in-person dual-task training is to be provided, incorporating more physical components could be beneficial. Similarly, any modified training approaches should undergo evaluation before being integrated into routine services. Future research studies could involve including a usual care control group or a waitlist control group to accurately determine the true impact of the training, building upon the improvement areas identified in this study. For instance, potential strategies for future interventions may involve implementing a 6-week intervention period, as some participants in the outcome evaluations exhibited positive effects as early as 6 weeks after the intervention; combining both in-person and online training modalities; and incorporating booster sessions. The exclusion criterion regarding engagement in any form of cognitive training 3 months before or during the study period may pose challenges once activities return to normal after the pandemic. Therefore, future trials should take a pragmatic approach, ensuring that any benefits derived from the intervention are in addition to participants’ existing daily lifestyle routines.

### Strengths and Limitations

The strength of this study included the integration of both quantitative and qualitative outcomes, which would reflect the feasibility and perceived benefits more holistically. The multiple outcome domains also helped to examine the potential effects more comprehensively. The multiple assessment points (4 time points) would facilitate the exploration of potential effects relative to the dosage of the intervention. The innovation components of the interventions were not restricted to the local context but could largely be applied to other regions.

Nevertheless, there were some limitations. The use of an attention control group might have limited the examination of potential effects from the intervention. Moreover, COVID-19 infection among the participants or their family members and the overall social environment during the study period were not documented, limiting the ability to control for the influences from these factors on the study outcomes. The cognitive domains assessed were limited to attention, verbal fluency, orientation, working memory, and executive function. Other cognitive domains, such as perceptual-motor control and social cognition, that might benefit from the intervention were not evaluated. User satisfaction was based on a self-developed question, and a validated scale could be considered in future. Owing to the nature of the feasibility study, the sample size was small, and a proper trial is needed in the future involving larger sample size.

### Conclusions

Online dual-task training was shown to be a feasible intervention and was well received by older adults during the pandemic. Within-group improvement was observed in subjective memory complaints and cognitive function, with a medium-to-large effect size in the intervention group. Further investigation in a full-scale RCT is required to fully explore the intervention’s potential effects.

## Appendix

Multimedia Appendix 1 Within-group and between-group differences in effect-related outcomes based on mixed effects model.

Multimedia Appendix 2 CONSORT eHEALTH checklist (V 1.6.1).

## Abbreviations

MIC: Memory Inventory in Chinese
HK-MoCA 5-Min: Montreal Cognitive Assessment 5 Minutes (Hong Kong Version)
PHQ-9: Patient Health Questionnaire-9
RCT: randomized controlled trial
