# Supplementary material for: Online Group–Based Dual-Task Training to Improve Cognitive Function of Community-Dwelling Older Adults: Randomized Controlled Feasibility Study
Source: JMIR Aging. 2025 May 16;8:e67267. doi: 10.2196/67267 (PMC12125557; doi:10.2196/67267)
Supplement: Multimedia Appendix 1 [file aging_v8i1e67267_app1.pdf]

Appendix 1a. Within-group difference in effect-related outcomes based on mixed effects model.

Outcomes	Intervention (n=50)			Control (n=26)		
	Values, mean[a] (SD)	Change from baseline[b] (95% CI)	Change from baseline, effect size[c] (95% CI)	Values, mean[a] (SD)	Change from baseline[b] (95% CI)	Change from baseline, effect size[c] (95% CI)
**Memory Inventory in Chinese[d]**						
Baseline	13.28 (3.86)	—[e]	—	11.03 (3.5)	—	—
Mid-intervention	10.76 (3.86)	−2.52 (−5.56 to 0.52)	−0.65 (−1.44 to 0.14)	9.06 (3.5)	−1.97 (−6.20 to 2.25)	−0.51 (−1.61 to 0.58)
Post-intervention	11.63 (3.86)	−1.65 (−5.35 to 2.05)	−0.43 (−1.39 to 0.53)	9.59 (3.5)	−1.44 (−6.70 to 3.81)	−0.37 (−1.74 to 0.99)
Follow-up	9.99 (3.86)	−3.29[f] (−6.15 to −0.42)	−0.85 (−1.59 to −0.11)	8.02 (3.5)	−3.01 (−7.05 to 1.03)	−0.78 (−1.83 to 0.27)
**Montreal Cognitive Assessment 5 Minutes (Hong Kong Version)**						
Baseline	26.64 (1.41)	—	—	25.85 (1.34)	—	—
Mid-intervention	27.08 (1.41)	0.44 (−0.46 to 1.34)	0.31 (−0.32 to 0.94)	26.51 (1.34)	0.67 (−0.58 to 1.91)	0.47 (−0.41 to 1.34)
Post-intervention	28.18 (1.41)	1.54[f] (0.71 to 2.38)	1.08 (0.49 to 1.66)	27.73 (1.34)	1.88[f] (0.69 to 3.07)	1.32 (0.48 to 2.15)

	Follow-up	27.73 (1.41)	1.09 (−0.17 to 2.34)	0.76 (−0.12 to 1.64)	28.04 (1.34)	2.20[f] (0.44 to 3.95)	1.54 (0.31 to 2.76)
**Digit Span Test—forward**							
	Baseline	6.66 (0.31)	—	—	6.80 (0.25)	—	—
	Mid-intervention	6.64 (0.31)	−0.02 (−0.28 to 0.23)	−0.08 (−0.94 to 0.78)	6.92 (0.25)	0.12 (−0.23 to 0.48)	0.41 (−0.78 to 1.59)
	Post-intervention	6.6 (0.31)	−0.06 (−0.34 to 0.21)	−0.21 (−1.14 to 0.71)	6.66 (0.25)	−0.14 (−0.54 to 0.26)	−0.46 (−1.79 to 0.87)
	Follow-up	6.57 (0.31)	−0.09 (−0.32 to 0.14)	−0.3 (−1.06 to 0.46)	6.89 (0.25)	0.09 (−0.23 to 0.41)	0.31 (−0.76 to 1.38)
**Digit Span Test—backward**							
	Baseline	4.03 (0.62)	—	—	3.84 (0.69)	—	—
	Mid-intervention	4.06 (0.62)	0.04 (−0.58 to 0.66)	0.05 (−0.91 to 1.02)	3.75 (0.69)	−0.09 (−0.95 to 0.77)	−0.14 (−1.48 to 1.19)
	Post-intervention	4.48 (0.62)	0.45 (−0.13 to 1.03)	0.70 (−0.20 to 1.60)	4.04 (0.69)	0.2 (−0.63 to 1.03)	0.31 (−0.98 to 1.59)
	Follow-up	4.02 (0.62)	0 (−0.61 to 0.60)	−0.01 (−0.95 to 0.93)	4.14 (0.69)	0.3 (−0.55 to 1.15)	0.47 (−0.86 to 1.79)
**Victoria Stroop Test—inference score for word[d]**							
	Baseline	2.87 (1.14)	—	—	3.45 (0.97)	—	—
	Mid-intervention	3 (1.14)	0.13 (−1.86 to 2.12)	0.11 (−1.67 to 1.90)	3.14 (0.97)	−0.31 (−3.07 to 2.46)	−0.28 (−2.76 to 2.21)

	Post-intervention	3.16 (1.14)	0.29 (−1.59 to 2.17)	0.26 (−1.43 to 1.95)	2.13 (0.97)	−1.32 (−4.01 to 1.37)	−1.19 (−3.61 to 1.23)
	Follow−up	4.34 (1.14)	1.46 (−0.8 to 3.73)	1.32 (−0.72 to 3.35)	2.29 (0.97)	−1.16 (−4.32 to 2.01)	−1.04 (−3.89 to 1.81)
**Victoria Stroop Test—inference score for color[d]**							
	Baseline	8.99 (3.11)	—	—	5.99 (2.66)	—	—
	Mid-intervention	7.26 (3.11)	−1.73 (−4.66 to 1.2)	−0.53 (−1.42 to 0.37)	6.49 (2.66)	0.51 (−3.55 to 4.56)	0.15 (−1.08 to 1.39)
	Post-intervention	6.81 (3.11)	−2.17 (−5.71 to 1.37)	−0.66 (−1.74 to 0.42)	6.71 (2.66)	0.72 (−4.33 to 5.76)	0.22 (−1.32 to 1.76)
	Follow-up	7.53 (3.11)	−1.46 (−4.09 to 1.17)	−0.44 (−1.25 to 0.36)	7.36 (2.66)	1.37 (−2.34 to 5.09)	0.42 (−0.72 to 1.55)
**Time to finish—5-times Chair Stand Test[d, g]**							
	Baseline	9.74 (1.76)	—	—	11.46 (1.67)	—	—
	Mid-intervention	11.95 (1.76)	2.21 (−3.49 to 7.92)	1.16 (−1.84 to 4.16)	17.82 (1.67)	6.36(−1.64 to 14.36)	3.34 (−0.86 to 7.55)
	Post-intervention	9.06 (1.76)	−0.68 (−1.98 to 0.62)	−0.36 (−1.04 to 0.32)	10.26 (1.67)	−1.19 (−3.07 to 0.69)	−0.63 (−1.62 to 0.36)
	Follow-up	12.33 (1.76)	2.59 (−3.65 to 8.83)	1.36 (−1.92 to 4.65)	20.85 (1.67)	9.40[f] (0.26 to 18.52)	4.94 (0.14 to 9.74)
**Instrumental Activity of Daily Living score**							
	Baseline	7.7 (0.25)	—	—	7.89 (0.3)	—	—

	Mid-intervention	7.55 (0.25)	−0.15 (−0.38 to 0.09)	−0.52 (−1.34 to 0.3)	7.8 (0.3)	−0.09 (−0.41 to 0.23)	−0.31 (−1.44 to 0.82)
	Post-intervention	7.37 (0.25)	−0.32[f] (−0.63 to −0.02)	−1.13 (−2.20 to −0.07)	7.61 (0.3)	−0.28 (−0.72 to 0.16)	−0.98 (−2.52 to 0.55)
	Follow-up	7.41 (0.25)	−0.29[f] (−0.56 to −0.01)	−1 (−1.95 to −0.05)	7.75 (0.3)	−0.14 (−0.53 to 0.24)	−0.5 (−1.85 to 0.84)
**Subjective Happiness Scale**							
	Baseline	4.86 (0.45)	—	—	5.07 (0.47)	—	—
	Mid-intervention	4.67 (0.45)	−0.19 (−0.58 to 0.19)	−0.41 (−1.25 to 0.42)	5.15 (0.47)	0.08 (−0.45 to 0.62)	0.17 (−0.98 to 1.33)
	Post-intervention	4.78 (0.45)	−0.08 (−0.46 to 0.31)	−0.16 (−0.99 to 0.66)	5.01 (0.47)	−0.07(−0.62 to 0.48)	−0.14 (−1.33 to 1.04)
	Follow-up	4.79 (0.45)	−0.07 (−0.43 to 0.29)	−0.15 (−0.93 to 0.63)	4.98 (0.47)	−0.09 (−0.60 to 0.42)	−0.19 (−1.30 to 0.91)
**Patient Health Questionnaire-9[d]**							
	Baseline	3.52 (1.73)	—	—	2.83 (1.64)	—	—
	Mid-intervention	4.99 (1.73)	1.47 (−0.24 to 3.19)	0.86 (−0.14 to 1.85)	3.56 (1.64)	0.73 (−1.63 to 3.09)	0.42 (−0.95 to 1.80)
	Post-intervention	4.82 (1.73)	1.30 (−0.25 to 2.85)	0.75 (−0.15 to 1.66)	4.27 (1.64)	1.44 (−0.79 to 3.66)	0.83 (−0.46 to 2.13)
	Follow-up	4.13 (1.73)	0.61 (−0.83 to 2.04)	0.35 (−0.48 to 1.19)	3.21 (1.64)	0.38 (−1.66 to 2.41)	0.22 (−0.96 to 1.40)

**6-item Lubben Social Network Scale**							
	Baseline	12.67 (2.01)	—	—	13.46 (2.33)	—	—
	Mid-intervention	12.64 (2.01)	−0.02 (−1.82 to 1.77)	−0.01 (−0.85 to 0.83)	11.32 (2.33)	−2.14 (−4.64 to 0.35)	−1 (−2.16 to 0.16)
	Post-intervention	13.2 (2.01)	0.53 (−1.37 to 2.43)	0.25 (−0.64 to 1.13)	12.31 (2.33)	−1.15 (−3.86 to 1.55)	−0.54 (−1.80 to 0.72)
	Follow-up	11.61 (2.01)	−1.06 (−3.05 to 0.94)	−0.49 (−1.42 to 0.44)	11.85 (2.33)	−1.61 (−4.43 to 1.2)	−0.75 (−2.07 to 0.56)

[a]On the basis of predicted values from the mixed effects model.

[b]Adjusted by age, gender, education level, comorbidity, frailty status and physical activity status at baseline.

[c]Effect size based on Cohen's *d*.

[d] Lower value indicates better condition.

[e]Not applicable.

[f]*P*<.05.

[g]For participants who could not finish 5 stands within 1 minute, the time was recorded as 60 seconds.

Appendix 1b. Between-group difference in effect-related outcomes based on mixed effects model.

Outcomes	Intervention: control		Intervention–high attendance: Intervention–low attendance[a]	
	Difference[b] (95% CI)	Effect size[c] (95% CI)	Difference[b] (95% CI)	Effect size[c] (95% CI)
**Memory Inventory in Chinese [d]**				
Mid-intervention	0.77 (−2.31 to 3.84)	0.20 (−0.60 to 1.00)	−4.56[e] (−8.86 to −0.26)	−1.18 (−2.30 to −0.07)
Post-intervention	1.42 (−2.98 to 5.82)	0.37 (−0.77 to 1.51)	−5.9[e] (−11.32 to −0.47)	−1.53 (−2.93 to −0.12)
Follow-up	1.08 (−1.78 to 3.94)	0.28 (−0.46 to 1.02)	1.18 (−2.45 to 4.81)	0.31 (−0.64 to 1.25)
**Montreal Cognitive Assessment 5 Minutes (Hong Kong Version)**				
Mid-intervention	0.49 (−0.59 to 1.58)	0.34 (−0.41 to 1.10)	−0.53 (−1.86 to 0.81)	−0.37 (−1.30 to 0.56)
Post-intervention	0.33 (−0.62 to 1.28)	0.23 (−0.43 to 0.90)	−0.19 (−1.43 to 1.05)	−0.13 (−1.00 to 0.74)
Follow-up	−0.43 (−1.83 to 0.97)	−0.30 (−1.28 to 0.68)	−0.36 (−2.45 to 1.72)	−0.25 (−1.71 to 1.20)
**Digit Span Test—forward**				
Mid-intervention	−0.13 (−0.45 to 0.20)	−0.42 (−1.50 to 0.65)	0.28 (−0.22 to 0.78)	0.94 (−0.74 to 2.62)
Post-intervention	0.10 (−0.25 to 0.46)	0.35 (−0.84 to 1.54)	0.11 (−0.23 to 0.46)	0.38 (−0.78 to 1.55)
Follow-up	−0.16 (−0.47 to 0.15)	−0.53 (−1.58 to 0.51)	0.22 (−0.24 to 0.68)	0.72 (−0.82 to 2.26)
**Digit Span Test—backward**				
Mid-intervention	0.24 (−0.45 to 0.92)	0.37 (−0.70 to 1.43)	−0.52 (−1.43 to 0.40)	−0.80 (−2.23 to 0.62)
Post-intervention	0.35 (−0.32 to 1.02)	0.55 (−0.50 to 1.59)	−0.74 (−1.56 to 0.08)	−1.15 (−2.42 to 0.12)

	Follow-up	−0.32 (−1.08 to 0.43)	−0.50 (−1.68 to 0.68)	0.23 (−0.82 to 1.29)	0.36 (−1.28 to 2.00)
**Victoria Stroop Test—inference score for word[d]**					
	Mid-intervention	−0.32 (−2.03 to 1.38)	−0.29 (−1.83 to 1.24)	0.29 (−1.79 to 2.37)	0.26 (−1.61 to 2.13)
	Post-intervention	0.29 (−1.71 to 2.29)	0.26 (−1.54 to 2.06)	2.0[e] (0.23 to 3.79)	1.81 (0.21 to 3.41)
	Follow-up	1.69 (−0.80 to 4.19)	1.52 (−0.72 to 3.76)	1.17 (−2.48 to 4.81)	1.05 (−2.23 to 4.33)
**Victoria Stroop Test—inference score for color[d]**					
	Mid-intervention	−2.40 (−5.37 to 0.58)	−0.73 (−1.64 to 0.18)	−2.49 (−5.90 to 0.92)	−0.76 (−1.80 to 0.28)
	Post-intervention	−0.88 (−3.94 to 2.17)	−0.27 (−1.20 to 0.66)	1.33 (−2.27 to 4.93)	0.41 (−0.69 to 1.50)
	Follow-up	−2.79 (−6.02 to 0.45)	−0.85 (−1.84 to 0.14)	−0.40 (−4.29 to 3.48)	−0.12 (−1.31 to 1.06)
**Time to finish— 5 times Chair Stand Test[d, f]**					
	Mid-intervention	−4.85 (−12.40 to 2.70)	−2.55 (−6.52 to 1.42)	−2.24 (−10.95 to 6.48)	−1.18 (−5.76 to 3.41)
	Post-intervention	0.07 (−1.41 to 1.55)	0.04 (−0.74 to 0.82)	1.84 (−5.48 to 9.16)	0.97 (−2.88 to 4.82)
	Follow-up	−7.34 (−15.58 to 0.91)	−3.86 (−8.19 to 0.48)	1.06 (−5.89 to 8.01)	0.56 (−3.10 to 4.21)
**Instrumental Activity of Daily Living score**					
	Mid-intervention	−0.12 (−0.41 to 0.18)	−0.41 (−1.43 to 0.62)	−0.19 (−0.62 to 0.23)	−0.68 (−2.17 to 0.82)
	Post-intervention	−0.12 (−0.52 to 0.28)	−0.43 (−1.84 to 0.97)	0.05 (−0.46 to 0.57)	0.19 (−1.60 to 1.99)
	Follow-up	−0.24 (−0.60 to 0.11)	−0.86 (−2.11 to 0.39)	0.07 (−0.48 to 0.62)	0.25 (−1.68 to 2.18)
**Subjective Happiness Scale**					

	Mid-intervention	−0.41 (−0.88 to 0.06)	−0.88 (−1.89 to 0.14)	0.39 (−0.22 to 1.01)	0.85 (−0.48 to 2.18)
	Post-intervention	−0.17 (−0.65 to 0.32)	−0.36 (−1.41 to 0.69)	−0.25 (−0.93 to 0.43)	−0.54 (−2.00 to 0.92)
	Follow-up	−0.13 (−0.62 to 0.36)	−0.28 (−1.34 to 0.78)	−0.03 (−0.70 to 0.63)	−0.07 (−1.50 to 1.36)
**Patient Health Questionnaire-9[d]**					
	Mid-intervention	0.79 (−1.34 to 2.92)	0.46 (−0.78 to 1.70)	−0.57 (−3.37 to 2.24)	−0.33 (−1.96 to 1.30)
	Post-intervention	0.29 (−1.60 to 2.19)	0.17 (−0.93 to 1.27)	−2.7[e] (−4.88 to −0.53)	−1.57 (−2.84 to −0.31)
	Follow-up	0.52 (−1.24 to 2.28)	0.30 (−0.72 to 1.33)	−0.21 (−2.71 to 2.28)	−0.12 (−1.57 to 1.33)
**6-item Lubben Social Network Scale**					
	Mid-intervention	1.99 (−0.15 to 4.12)	0.93 (−0.07 to 1.92)	−1.19 (−3.76 to 1.39)	−0.56 (−1.76 to 0.65)
	Post-intervention	1.23 (−1.07 to 3.53)	0.57 (−0.50 to 1.65)	−2.76 (−5.54 to 0.03)	−1.29 (−2.59 to 0.02)
	Follow-up	0.39 (−2.08 to 2.87)	0.18 (−0.97 to 1.34)	−2.08 (−5.62 to 1.46)	−0.97 (−2.62 to 0.68)

[a]High attendance refers to attendance ≥75% and low attendance refers to attendance <75%.

[b]Adjusted by age, gender, education level, comorbidity, frailty status, physical activity status and the corresponding outcome at baseline.

[c]Effect size based on Cohen's *d.*

[d]Lower value indicates better condition.

[e]*P*<.05.

[f]For participants who could not finish 5 stands within 1 minute, the time was recorded as 60 seconds.
